# Cross-sectional association between serum concentrations of *n*-3 long-chain PUFA and depressive symptoms: results in Japanese community dwellers

**DOI:** 10.1017/S0007114515004754

**Published:** 2015-12-22

**Authors:** Chika Horikawa, Rei Otsuka, Yuki Kato, Yukiko Nishita, Chikako Tange, Saki Kakutani, Tomohiro Rogi, Hiroshi Kawashima, Hiroshi Shibata, Fujiko Ando, Hiroshi Shimokata

**Affiliations:** 1 Section of the NILS-LSA, National Center for Geriatrics and Gerontology, Aichi 474-8511, Japan; 2 Institute for Health Care Science, Suntory Wellness Limited, Kyoto 619-0284, Japan; 3 Faculty of Health and Medical Sciences, Aichi Shukutoku University, Aichi 480-1197, Japan; 4 Graduate School of Nutritional Sciences, Nagoya University of Arts and Sciences, Aichi 470-0196, Japan

**Keywords:** *n*-3 Long-chain PUFA, Depressive symptoms, Community-dwelling people, Japanese

## Abstract

The effect of *n*-3 long-chain PUFA (*n*-3 LCPUFA) on depression in healthy subjects is unclear, and most of the previous studies have focused on populations eating Western diets with lower fish intake. The present study investigated the association between blood levels of *n*-3 LCPUFA and depressive symptoms in Japanese community dwellers with higher *n*-3 LCPUFA blood levels. A cross-sectional study was conducted from 2006 to 2008, including 1050 men and 1073 women aged 40 years or older from the National Institute for Longevity Sciences – the Longitudinal Study of Aging. The Center for Epidemiologic Studies Depression Scale (CES-D) was used to assess depressive symptoms. Multiple logistic regression analysis was performed to estimate the OR and 95 % CI for a CES-D score ≥16. Serum concentrations of *n*-3 PUFA, but not *n*-6 PUFA, were inversely associated with depressive symptoms. Compared with the lowest quintile, the adjusted OR for serum EPA at the fourth and fifth quintiles were 0·55 (95 % CI 0·35, 0·85) and 0·64 (95 % CI 0·42, 0·98), respectively, and at the fifth quintile for DHA it was 0·58 (95 % CI 0·37, 0·92), for the presence of depressive symptoms (*P*_for trend_=0·013 and 0·011, respectively). Serum levels of EPA and DHA were inversely associated with depressive symptoms in Japanese community dwellers with higher blood levels of *n*-3 LCPUFA, suggesting that *n*-3 LCPUFA intakes corresponding to higher levels in a Japanese population may have implications for a lower prevalence of depression.

Depression is a serious public health problem worldwide^(^ ^1^ ^)^. According to the seventeen nation World Mental Health Survey, approximately 5 % of people report having experienced at least one episode of depression during their life^(^ ^2^ ^)^. By 2030, depression is predicted to become one of the top three causes of disability-adjusted life years lost^(^ ^3^ ^)^.

Management and prevention of depression are becoming increasingly important, especially for the middle-aged and the elderly. Depression in the middle-aged population results in economic losses and social burden; this social burden arises because the roles of middle-aged people in a community, including working and child rearing, keep growing. In addition, depressive symptoms are inter-related with multiple chronic conditions and age-related cognitive impairment^(^ ^4^ ^)^, as well as prodromal signs of cognitive decline in patients with early dementia^(^ ^5^ ^,^ ^6^ ^)^. Thus, depression has attracted attention as a potential risk factor for dementia. Therefore, it should be recognised as a problem that needs to be addressed immediately in developed countries with ageing societies.

It is well known that diet and nutrition influence depression. EPA and DHA, the two major *n*-3 long-chain PUFA (*n*-3 LCPUFA) derived from fish, are essential for the maintenance of cellular membrane functions via determination of membrane fluidity, and they affect neurotransmitter release and modify receptor binding^(^ ^7^ ^)^. Previous experimental studies have shown various mechanisms of these fatty acids, including anti-inflammatory^(^ ^8^ ^)^, antioxidative^(^ ^9^ ^)^, neuroprotective^(^ ^10^ ^)^ and neurogenesis effects^(^ ^11^ ^)^. These factors may also be important in protecting against depression and its symptoms^(^ ^12^ ^)^. Thus, we hypothesised that *n*-3 LCPUFA may be able to influence the pathophysiology of depression.

According to a recent meta-analysis that took into account the heterogeneity among populations of randomised clinical trials, the use of *n*-3 LCPUFA was effective in patients with major depressive disorder (MDD) and in depressed patients without a diagnosis of MDD^(^ ^13^ ^)^. A meta-analysis of case–control studies, using blood levels as an indicator of *n*-3 LCPUFA levels, also reported significantly lower levels of EPA, DHA and total *n*-3 LCPUFA to be associated with depression^(^ ^14^ ^)^. These findings of associations in a general population are important in terms of preventing depression.

Previous ecological studies have shown that countries with high fish consumption, such as Japan, were associated with lower age-standardised disability-adjusted life-year rates for depressive disorders^(^ ^15^ ^)^. However, studies on markers of dietary intake of *n*-3 LCPUFA in the same area/region provided contrasting results. To the best of our knowledge, only a few observational studies have examined the association between depression and *n*-3 LCPUFA, evaluated by measuring blood samples or intake estimation, in a general population with relatively high blood levels of *n*-3 LCPUFA^(^ ^16^ ^,^ ^17^ ^)^. The lack of evidence regarding depressive symptoms in countries with high fish consumption may be explained in part by levels of *n*-3 LCPUFA being high enough to interact with the expression of depression.

The aim of our study was to investigate the association between blood levels of *n*-3 LCPUFA and depressive symptoms in a cross-sectional analysis of a community-dwelling population with higher *n*-3 LCPUFA blood levels. We specifically focused on middle-aged and elderly people, who have a higher risk for depression. We also examined other fatty acids comparatively, because it is necessary to ascertain whether findings regarding any association are specific to *n*-3 LCPUFA.

## Methods

Data for the present study were obtained from the National Institute for Longevity Sciences – the Longitudinal Study of Aging (NILS-LSA), a population-based survey of ageing in Japan. The participants in the NILS-LSA include about 2300 selected middle-aged and elderly subjects^(^ ^18^ ^)^. The normal ageing process has been researched over time using detailed questionnaires and medical check-ups, anthropometric measurements, physical fitness tests and nutritional assessments. Participants in the NILS-LSA included randomly selected age- and sex-stratified individuals from the pool of non-institutionalised residents of the National Center for Geriatrics and Gerontology’s neighbourhood areas of Obu City and Higashiura Town in Aichi Prefecture. Details of the NILS-LSA study have been reported elsewhere^(^ ^18^ ^)^.

### Subjects

**Fig. 1:**
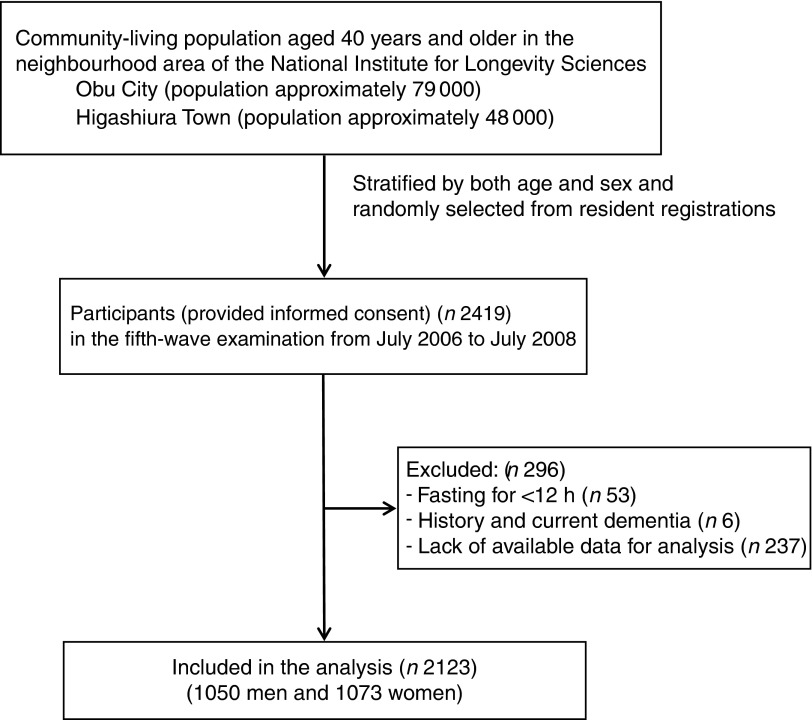
Flow chart for the individuals included in the study.

Subjects in this cross-sectional analysis were derived from the fifth-wave examination of the NILS-LSA. The fifth-wave examination, which comprehensively measured various fatty acids, comprised 2419 participants aged 40 years and older, and was started in July 2006 and completed in July 2008. After the exclusion of 296 subjects because of fasting for <12 h (*n* 53), history of and current dementia (*n* 6) or lack of available data for analysis (*n* 237), a total of 2123 subjects (1050 men, 1073 women) were included in the analysis (Fig. 1). Self-reported history of previously diagnosed dementia was collected at the fifth-wave interview.

The study was approved by the Committee for Ethics of Human Research of the National Center for Geriatrics and Gerontology (no. 249). Written informed consent was obtained from all the subjects.

### Serum fatty acid levels

Venous blood was collected early in the morning after at least 12 h of fasting. Blood samples were centrifuged at 3500 ***g*** for 15 min. Serum was separated and immediately frozen at −80°C. Total lipids in serum were extracted and purified by the Folch procedure. Serum fatty acid concentration was measured using a GC at a clinical laboratory (SRL). In brief, fatty acid residues in the lipids were transmethylated and analysed using a GC (GC-17A; Shimadzu) with a capillary column Omegawax 250 (Supelco). Absolute amounts of each fatty acid were quantified by standards as weight concentrations (μg/ml).

### Depressive symptoms

Depressive symptoms were assessed using the Japanese version^(^ ^19^ ^)^ questionnaire of the Center for Epidemiologic Studies Depression Scale (CES-D)^(^ ^20^ ^)^. Participants completed a CES-D questionnaire, which assessed the depressed state during a previous week, approximately 2 weeks before each exploratory visit for the fifth-wave examination. The CES-D questionnaire comprises twenty questions in four subscales: somatic and retarded activity, depressed affect, positive affect and interpersonal relations. Scores range from 0 to 60, with lower scores indicating fewer depressive symptoms. This scale has been reported to be a valid and reliable measure of depressive symptoms in the elderly^(^ ^21^ ^)^. A cut-off score of ≥16 is used to identify subjects with relevant depressive symptoms^(^ ^20^ ^)^. We defined a CES-D score ≥16 as representative of depressive symptoms.

### Other measurements

Data regarding medical history of hypertension, hyperlipidaemia, ischaemic heart disease, stroke, diabetes and dementia (yes/no), education (≤9, 10–12, or ≥13 years of school), employment status (no occupation or household labour/non-regular employment/regular employment), marital status (yes/no), sex, age and smoking status (yes/no) were asked in the survey questionnaires. This information was obtained via a self-completed questionnaire provided approximately 2 weeks before the exploratory visit; questionnaires were collected on the same day as blood samples. BMI was calculated as weight in kilograms divided by the square of height in metres (kg/m^2^). Both weight and height were actual measured values obtained on the same day that blood samples were collected. Alcohol intake (ml/d) during the previous year was assessed using a FFQ through interviews with well-trained dietitians. The number of steps walked per day was used to assess physical activity. Participants wore a uniaxial accelerometry sensor (Lifecorder; Suzuken) for a 7-d period (except while sleeping or bathing) for 2 weeks after the exploratory visit. We calculated the mean number of steps from 5-d records (the maximum and minimum values were excluded).

### Statistical analysis

All the statistical analyses were conducted using Statistical Analysis System version 9.3 (SAS Institute). The confounding variables were sex, age, BMI, education level, marital status, smoking status, alcohol consumption, physical activity, employment status and history of hypertension, hyperlipidaemia, ischaemic heart disease, stroke and diabetes. Differences in characteristics and serum fatty acid concentrations between subjects with and without depressive symptoms were assessed using the *χ* ^2^ test (categorical variables) and Student’s *t* test (continuous variables). For analysis of the association between serum fatty acid concentrations and depressive symptoms, we carried out multiple logistic regression analysis and estimated the OR and 95 % CI of depressive symptoms for quintiles of serum fatty acid concentrations, using the lowest quintile category as the reference. Trend associations were assessed by entering dummy variables assigned to the quintile of serum fatty acid concentrations. In model I, we adjusted for age and sex. Model II was further adjusted for the above-described confounding variables. A sub-analysis by sex also was performed for EPA and DHA. Two-sided *P* values<0·05 were regarded as statistically significant.

## Results

The study subjects included 1050 men (49·5 %) and 1073 women (50·5 %). In total, the mean age was 60·3 (sd 12·3) years, and 266 subjects (12·5 %) were identified as having depressive symptoms (CES-D scores ≥16). Characteristics according to subjects with and without depressive symptoms are shown in Table 1. Subjects with depressive symptoms were significantly more likely to be unmarried, have fewer years of education and have a higher rate of past stroke compared with subjects without depressive symptoms.

**Table 1:** Characteristics of subjects with and without depressive symptoms (Numbers and percentages; mean values and standard deviations)

	Subjects without depressive symptoms (CES-D<16)		Subjects without depressive symptoms (CES-D≥16)		
Variables	*n*	%	*n*	%	*P* *
Number of subjects	1857		266		
Sex (male)	933	50·2	117	44·0	0·056
Age (years)					0·122
Mean	60·1		61·4		
sd	12·3		12·8		
BMI (kg/m^2^)					0·071
Mean	22·9		22·5		
sd	3·0		3·2		
CES-D					<0·001
Mean	5·3		21·9		
sd	4·4		6·2		
Education level					
≤9 years	329	17·7	58	21·8	0·038
10–12 years	738	39·7	116	43·6	
≥13 years	790	42·5	92	34·6	
Unmarried	254	13·7	72	27·1	<0·001
Current smoker	262	14·1	45	16·9	0·223
Alcohol consumption† (ml/d)					0·842
Mean	0·37		0·35		
sd	2·61		1·96		
Physical activity (steps/d)					0·274
Mean	8746		8500		
sd	3417		3439		
Employment					
Unemployed or household labour	766	41·3	123	46·2	0·207
Non-regular employment	385	20·7	56	21·1	
Regular employment	706	38·0	87	32·7	
Medical history‡					
Stroke	53	2·9	16	6·0	0·007
Hypertension	513	27·6	82	30·8	0·277
Ischaemic heart disease	74	4·0	13	4·9	0·488
Hyperlipidaemia	349	18·8	59	22·2	0·190
Diabetes mellitus	134	7·2	21	7·9	0·691

CES-D, Center for Epidemiologic Studies Depression Scale.

* Continuous variables: Student’s *t* test, Categorical variables: *χ* ^2^ test.

† Converting alcohol consumption into ethanol content.

‡ Past and present illness.

Serum concentrations of principal fatty acids based on the presence or absence of depressive symptoms are shown in Table 2. There were no significant differences in any serum fatty acid concentrations between groups. However, several fatty acids showed marginally significant differences between subjects with and without depressive symptoms, including EPA (*P*=0·068), *n*-3 PUFA (*P*=0·072) and *n*-3 LCPUFA (*P*=0·078). The mean *n*-3 LCPUFA concentrations of subjects with and without depressive symptoms were 264·1 (sd 101·2) μg/ml and 276·0 (sd 103·4) μg/ml, respectively.

**Table 2:** Serum fatty acid concentration (μg/ml) and ratio of study subjects (Mean values and standard deviations)

	Subjects without depressive symptoms (CES-D<16)		Subjects with depressive symptoms (CES-D≥16)		
	Mean	sd	Mean	sd	*P* *
SFA	1001·8	270·6	991·9	254·9	0·571
MUFA	707·3	260·9	710·9	222·1	0·807
PUFA	1454·2	274·9	1429·7	250·7	0·170
*n*-3 PUFA	303·9	110·6	290·9	105·9	0·072
DHA	174·0	58·4	168·2	57·3	0·125
EPA	78·8	44·1	73·5	43·4	0·068
ALA	27·9	19·5	26·8	12·2	0·218
*n*-6 PUFA	1148·7	222·5	1137·1	199·6	0·385
ARA	174·7	40·1	174·0	39·5	0·786
LA	915·8	192·0	905·2	174·1	0·359
*n*-3:*n*-6	0·270	0·101	0·260	0·095	0·143

CES-D, Center for Epidemiologic Studies Depression Scale; *n*-3 PUFA, sum of ALA, EPA, DPA and DHA; ALA, *α*-linolenic acid; *n*-6 PUFA, sum of LA, *γ*-linolenic acid, eicosadienoic acid, dihomo-*γ*-linolenic acid, ARA and docosatetraenoic acid; ARA, arachidonic acid; LA, linoleic acid.

* Student’s *t* test.

Tables 3 and 4 present the OR and 95 % CI of depressive symptoms for quintiles of serum fatty acid concentrations, using the lowest quintile category as the reference. In Table 3, SFA, MUFA, PUFA and *n*-6 PUFA were not significant, but the *n*-3:*n*-6 ratio partially and *n*-3 PUFA showed an inverse association in all models. Individual fatty acids of the PUFA series are shown in Table 4. In the crude model, EPA and DHA showed an inverse association with depressive symptoms (*P*_for trend_=0·021 and 0·039, respectively). The crude OR for EPA at the fourth quintile was lower than that for the lowest quintile (OR 0·58; 95 % CI 0·38, 0·88). In Model I, adjusting for sex and age, the OR for EPA at the fourth and fifth quintiles and for DHA at the fifth quintile were significantly lower compared with reference values. Furthermore, these significant associations were maintained, even after adjusting for more covariates including age, sex, BMI, education level, marital status, smoking status, alcohol consumption, physical activity, employment status and history of hypertension, hyperlipidaemia, ischaemic heart disease, stroke and diabetes. In Model II, after adjusting for all covariates, EPA was associated with a lower OR of depressive symptoms (fourth quartile *v*. lowest: OR 0·55; 95 % CI 0·35, 0·85; fifth quartile *v*. lowest: OR 0·64; 95 % CI 0·42, 0·98; *P*_for trend_=0·013). Similarly, the quintile of DHA was associated with a lower OR of depressive symptoms (fifth quartile *v*. lowest: OR 0·58; 95 % CI 0·37, 0·92; *P*_for trend_=0·011). There were no significant associations for other fatty acids. In addition, a sub-analysis by sex was performed for EPA and DHA. In women only, EPA showed a significant association with a lower OR of depressive symptoms in model II (fourth quartile *v*. lowest: OR 0·47; 95 % CI 0·26, 0·86; fifth quartile *v*. lowest: OR 0·51; 95 % CI 0·28, 0·93; *P*_for trend_=0·013). There were no significant associations for DHA in both sexes.

**Table 3:** Depressive symptoms according to quintile of serum fatty acid concentration and ratio (Odds ratios and 95 % confidence intervals)

	Q1 (low)		Q2		Q3		Q4		Q5 (high)		
	OR	95 % CI	OR	95 % CI	OR	95 % CI	OR	95 % CI	OR	95 % CI	*P*_for trend_ †
SFA											
Median of serum fatty acid concentration (μg/ml)	739·9		857·3		950·4		1072·3		1302·9		
Range of serum fatty acid concentration (μg/ml)	456·7–806·2		806·3–902·1		902·3–1005·0		1005·1–1155·2		1155·5–4234·4		
Number with/without depressive symptoms‡	51/373		54/369		63/363		44/381		54/371		
Crude	1·00	Ref.	1·07	0·71, 1·61	1·27	0·85, 1·89	0·85	0·55, 1·30	1·07	0·71, 1·60	0·812
Model I: multivariate-adjusted§	1·00	Ref.	1·04	0·69, 1·57	1·19	0·80, 1·78	0·80	0·52, 1·23	1·04	0·69, 1·57	0·691
Model II: Multivariate-adjusted||	1·00	Ref.	1·05	0·69, 1·59	1·18	0·79, 1·78	0·80	0·52, 1·25	1·04	0·67, 1·60	0·698
MUFA											
Median of serum fatty acid concentration (μg/ml)	481·9		572·8		653·0		762·4		982·0		
Range of serum fatty acid concentration (μg/ml)	298·3–533·6		533·8–611·2		611·6–702·1		702·5–839·4		839·5–4727·5		
Number with/without depressive symptoms‡	50/374		51/374		50/374		60/365		55/370		
Crude	1·00	Ref.	1·02	0·67, 1·55	1·00	0·66, 1·52	1·23	0·82, 1·84	1·11	0·74, 1·67	0·391
Model I: multivariate-adjusted§	1·00	Ref.	0·99	0·66, 1·51	0·95	0·62, 1·45	1·18	0·78, 1·77	1·10	0·73, 1·66	0·446
Model II: multivariate-adjusted||	1·00	Ref.	1·01	0·66, 1·54	0·96	0·62, 1·47	1·17	0·77, 1·79	1·06	0·69, 1·65	0·582
PUFA											
Median of serum fatty acid concentration (μg/ml)	1150·7		1301·0		1427·3		1550·6		1779·1		
Range of serum fatty acid concentration (μg/ml)	666·8–1233·4		1233·5–1369·5		1369·5–1487·4		1488·1–1647·1		1648·0–4618·2		
Number with/without depressive symptoms‡	58/366		55/370		60/364		46/379		47/378		
Crude	1·00	Ref.	0·95	0·63, 1·43	1·02	0·68, 1·52	0·75	0·49, 1·14	0·80	0·52, 1·22	0·143
Model I: multivariate-adjusted§	1·00	Ref.	0·93	0·62, 1·38	0·99	0·67, 1·47	0·74	0·49, 1·12	0·77	0·51, 1·16	0·110
Model II: multivariate-adjusted||	1·00	Ref.	0·94	0·63, 1·39	1·04	0·71, 1·54	0·77	0·51, 1·16	0·79	0·52, 1·18	0·154
*n*-3 PUFA											
Median of serum fatty acid concentration (μg/ml)	182·1		237·4		288·2		341·1		436·5		
Range of serum fatty acid concentration (μg/ml)	77·5–210·5		210·7–262·5		262·6–310·8		311·0–379·8		379·9–1197·5		
Number with/without depressive symptoms‡	59/365		62/362		51/373		54/372		40/385		
Crude	1·00	Ref.	1·06	0·72, 1·56	0·85	0·57, 1·26	0·90	0·60, 1·34	0·64*	0·42, 0·98	0·028*
Model I: multivariate-adjusted§	1·00	Ref.	0·99	0·67, 1·47	0·76	0·50, 1·15	0·79	0·52, 1·19	0·55*	0·35, 0·86	0·005**
Model II: multivariate-adjusted||	1·00	Ref.	0·99	0·66, 1·47	0·75	0·49, 1·15	0·77	0·51, 1·18	0·57*	0·36, 0·90	0·009**
*n*-6 PUFA											
Median of serum fatty acid concentration (μg/ml)	892·0		1030·7		1130·3		1238·1		1413·2		
Range of serum fatty acid concentration (μg/ml)	580·6–969·1		969·3–1079·6		1079·9–1178·0		1178·2–1310·4		1310·8–3412·8		
Number with/without depressive symptoms‡	56/368		51/374		56/368		53/372		50/375		
Crude	1·00	Ref.	0·90	0·60, 1·35	1·00	0·67, 1·49	0·94	0·63, 1·40	0·88	0·58, 1·32	0·636
Model I: multivariate-adjusted§	1·00	Ref.	0·90	0·60, 1·35	0·99	0·66, 1·48	0·92	0·61, 1·39	0·90	0·59, 1·35	0·676
Model II: multivariate-adjusted||	1·00	Ref.	0·89	0·59, 1·35	1·00	0·66, 1·51	0·94	0·62, 1·42	0·90	0·59, 1·38	0·753
*n*-3:*n*-6											
Median of ratio	0·160		0·209		0·250		0·303		0·401		
Range of ratio	0·078–0·187		0·187–0·231		0·231–0·271		0·271–0·336		0·336–0·962		
Number with/without depressive symptoms‡	60/364		57/368		52/372		42/383		55/370		
Crude	1·00	Ref.	0·94	0·64, 1·39	0·85	0·57, 1·26	0·67	0·44, 1·01	0·90	0·61, 1·34	0·226
Model I: multivariate-adjusted§	1·00	Ref.	0·88	0·59, 1·31	0·76	0·50, 1·15	0·58*	0·38, 0·91	0·77	0·50, 1·18	0·063
Model II: multivariate-adjusted||	1·00	Ref.	0·90	0·60, 1·35	0·76	0·50, 1·16	0·58*	0·37, 0·91	0·78	0·50, 1·20	0·066

Q, quintiles; Ref., referent value; *n*-3 PUFA, sum of *α*-linolenic acid, EPA, DPA and DHA; *n*-6 PUFA, sum of linoleic acid, gamma-linolenic acid, eicosadienoic acid, dihomo-*γ*-linolenic acid, arachidonic acid and docosatetraenoic acid. * *P*<0·05, ** *P*<0·01.

† *P* for trend estimated by treating quintiles as ordinal variables for serum fatty acid concentration.

‡ A person with total score ≥16 using the Center for Epidemiologic Studies Depression Scale is supposed to have a significant depressive tendency.

§ Model I: adjusted for sex and age.

|| Model II: adjusted for sex, age, BMI, education level, marital status, smoking status, alcohol consumption, physical activity, employment status and medical history.

**Table 4:** Depressive symptoms according to quintile of serum fatty acid concentration (Odds ratios and 95 % confidence intervals)

	Q1 (low)		Q2		Q3		Q4		Q5 (high)		
	OR	95 % CI	OR	95 % CI	OR	95 % CI	OR	95 % CI	OR	95 % CI	*P*_for trend_ †
*n*-3 PUFA											
DHA											
Median of serum fatty acid concentration (μg/ml)	108·8		138·5		165·6		195·0		244·9		
Range of serum fatty acid concentration (μg/ml)	25·8–125·0		125·2–153·2		153·3–178·4		178·5–214·2		214·4–609·3		
Number with/without depressive symptoms‡	56/368		64/361		52/372		55/368		39/388		
Crude	1·00	Ref.	1·17	0·79, 1·72	0·92	0·61, 1·38	0·98	0·66, 1·46	0·66	0·43, 1·02	0·039*
Model I: multivariate-adjusted§	1·00	Ref.	1·10	0·74, 1·62	0·84	0·55, 1·27	0·87	0·58, 1·31	0·57*	0·36, 0·90	0·008**
Model II: multivariate-adjusted||	1·00	Ref.	1·09	0·74, 1·63	0·80	0·53, 1·23	0·86	0·56, 1·31	0·58*	0·37, 0·92	0·011*
EPA											
Median of serum fatty acid concentration (μg/ml)	31·9		51·5		69·1		92·2		131·2		
Range of serum fatty acid concentration (μg/ml)	10·1–41·6		41·8–59·9		60·0–79·6		79·7–107·1		107·3–333·1		
Number with/without depressive symptoms‡	66/354		54/375		57/367		41/381		48/380		
Crude	1·00	Ref.	0·77	0·52, 1·14	0·83	0·57, 1·22	0·58*	0·38, 0·88	0·68	0·46, 1·01	0·021*
Model I: multivariate-adjusted§	1·00	Ref.	0·74	0·50, 1·09	0·78	0·53, 1·15	0·52*	0·34, 0·80	0·61	0·40, 0·93*	0·006**
Model II: multivariate-adjusted||	1·00	Ref.	0·77	0·52, 1·15	0·77	0·52, 1·15	0·55*	0·35, 0·85	0·64	0·42, 0·98*	0·013*
ALA											
Median of serum fatty acid concentration (μg/ml)	15·7		20·2		24·8		30·2		42·4		
Range of serum fatty acid concentration (μg/ml)	7·7–18·0		18·1–22·3		22·4–27·2		27·3–34·4		34·5–673·6		
Number with/without depressive symptoms‡	58/359		55/373		61/364		45/383		47/378		
Crude	1·00	Ref.	0·91	0·61, 1·36	1·04	0·70, 1·53	0·73	0·48, 1·10	0·77	0·51, 1·16	0·111
Model I: multivariate-adjusted§	1·00	Ref.	0·88	0·59, 1·31	0·99	0·67, 1·46	0·67	0·44, 1·02	0·74	0·49, 1·12	0·065
Model II: multivariate-adjusted||	1·00	Ref.	0·92	0·61, 1·38	1·00	0·67, 1·50	0·70	0·45, 1·08	0·73	0·48, 1·13	0·073
*n*-6 PUFA											
ARA											
Median of serum fatty acid concentration (μg/ml)	128·4		152·6		170·3		191·8		225·4		
Range of serum fatty acid concentration (μg/ml)	66·6–142·3		142·4–161·2		161·3–179·7		179·8–205·6		205·7–411·0		
Number with/without depressive symptoms‡	55/369		48/376		56/369		55/370		52/373		
Crude	1·00	Ref.	0·86	0·57, 1·29	1·02	0·68, 1·52	1·00	0·67, 1·49	0·94	0·62, 1·40	0·968
Model I: multivariate-adjusted§	1·00	Ref.	0·84	0·56, 1·27	1·00	0·67, 1·50	0·96	0·64, 1·44	0·94	0·63, 1·42	0·975
Model II: multivariate-adjusted||	1·00	Ref.	0·82	0·54, 1·25	1·00	0·66, 1·50	0·95	0·63, 1·44	0·93	0·61, 1·41	0·987
LA											
Median of serum fatty acid concentration (μg/ml)	698·3		809·2		899·2		944·9		1151·3		
Range of serum fatty acid concentration (μg/ml)	409·8–759·2		759·5–856·2		856·4–942·5		942·7–1053·1		1053·3–2839·5		
Number with/without depressive symptoms‡	53/371		57/368		53/371		53/372		50/375		
Crude	1·00	Ref.	1·08	0·73, 1·62	1·00	0·67, 1·50	1·00	0·66, 1·50	0·93	0·62, 1·41	0·636
Model I: multivariate-adjusted§	1·00	Ref.	1·11	0·74, 1·66	0·99	0·66, 1·49	0·99	0·66, 1·50	0·96	0·63, 1·46	0·691
Model II: multivariate-adjusted||	1·00	Ref.	1·10	0·73, 1·65	0·99	0·65, 1·51	1·00	0·66, 1·52	0·98	0·64, 1·50	0·783

Q, quintiles; Ref., referent value; ALA, *α*-linolenic acid; ARA, arachidonic acid; LA, linoleic acid. * *P*<0·05, ** *P*<0·01.

† *P*_for trend_ estimated by treating quintiles as ordinal variables for serum fatty acid concentration.

‡ A person with total score ≥16 using the Center for Epidemiologic Studies Depression Scale is supposed to have a significant depressive tendency.

§ Model I: adjusted for sex and age.

|| Model II: adjusted for sex, age, BMI, education level, marital status, smoking status, alcohol consumption, physical activity, employment status, and medical history.

## Discussion

In the present study, inverse associations between serum EPA and DHA concentrations and depressive symptoms were observed in Japanese middle-aged and elderly community-dwelling subjects. This is the first study demonstrating the inverse association in community-dwelling Japanese people with higher blood levels of *n*-3 LCPUFA. No positive association for other fatty acids indicates that this association is specific to *n*-3 LCPUFA.

The prevalence of depressive symptoms among subjects was 12·5 % in this study. In another survey of nearly 5000 Japanese elderly aged 65 years and older, Yokoyama *et al*.^(^ ^22^ ^)^ showed that the prevalence of depression was 13·8 %. The incidence rate is approximately the same as the present study, although depression in their study was evaluated according to the eleven-item short form of the CES-D.

Subjects in the present study were Japanese and had higher serum levels of *n*-3 LCPUFA compared with populations in Western countries^(^ ^23^ ^–^ ^25^ ^)^. Median values for serum EPA, DHA and *n*-3 LCPUFA were 69·1, 165·6 and 261·3 μg/ml, respectively. Japanese consume a large amount of fish and seafood^(^ ^26^ ^)^, the main sources of *n*-3 LCPUFA^(^ ^24^ ^)^, and *n*-3 LCPUFA intake correlates well with blood levels^(^ ^27^ ^)^. In a previous study, Japanese middle-aged and elderly subjects also showed similar levels of serum *n*-3 LCPUFA^(^ ^28^ ^)^. In American middle-aged and elderly subjects with hypercholesterolaemia (*n* 105), the median values for serum EPA and DHA were 18 and 46 μg/ml, respectively^(^ ^29^ ^)^. In Brazilian middle-aged subjects (<50 years), the median value for serum *n*-3 LCPUFA was 150 μg/ml^(^ ^30^ ^)^. Although not a stringent comparison because we determined non-fractionated and serum concentrations in our study, the mean compositions for erythrocyte phospholipids of EPA and DHA in another Japanese elderly population were 2·3 and 6·7 %, respectively^(^ ^31^ ^)^, and 0·3 and 3·7 % in elderly Italian females^(^ ^32^ ^)^. The *n*-3 LCPUFA blood level in Japanese subjects is more than two times higher compared with that of subjects from Western countries.

A recent meta-analysis was conducted in countries with populations with lower blood levels of *n*-3 LCPUFA, and the results showed lower blood levels of *n*-3 LCPUFA in patients with depression^(^ ^14^ ^)^. An inverse association between serum *n*-3 LCPUFA levels and depressive symptoms in community-dwelling people with higher blood levels of *n*-3 LCPUFA has not been observed so far. We considered that the lack of evidence regarding depressive symptoms in populations with higher blood levels of *n*-3 LCPUFA may depend on levels of *n*-3 LCPUFA being high enough to interact with the expression of depression. However, we found significant trends for these inverse associations between serum *n*-3 LCPUFA and depressive symptoms in the present study.

To the best of our knowledge, only two studies investigated the association between serum levels of *n*-3 LCPUFA and depressive symptoms in Korean or Japanese subjects with high blood levels of *n*-3 LCPUFA^(^ ^16^ ^,^ ^17^ ^)^. In the Japanese study, no association was observed between blood *n*-3 LCPUFA levels and depressive symptoms, whereas a significant association was noted in Korean subjects. The Korean study used a case–control design with eighty depressive patients and eighty controls, including a higher percentage of women. On the other hand, the Japanese study included 113 healthy men. The composition ratio of sex may partially influence results. According to three previous studies of dietary intervention in Western countries, the association appears to be observed more in women than in men^(^ ^33^ ^–^ ^35^ ^)^. The NILS-LSA is a cohort study that has approximately 280 subjects in each group classified by age decade and sex, and thus roughly half the subjects are women. We showed a significant association for EPA in women through a sub-analysis stratified by sex, and assumed higher endogenous *n*-3 LCPUFA in women because of oestrogenic effects as a reasonable possibility for this sex difference^(^ ^36^ ^)^. However, this finding should be interpreted with caution in light of evidence from a previous report implicating the stronger association in men than in women between *n*-3 LCPUFA intakes and the occurrence of depressive episodes^(^ ^37^ ^)^.

The Korean study was conducted in patients with depression^(^ ^17^ ^)^, but subjects in the Japanese study were healthy^(^ ^16^ ^)^. Most previous studies showing a significant association between *n*-3 LCPUFA and depressive symptoms were conducted in depressed patients^(^ ^13^ ^,^ ^14^ ^)^. However, subjects who participated in the NILS-LSA were non-hospitalised, community-dwelling people, and we did not have a diagnosis of clinical depression. There might be other reasons for our findings.

Our findings may be due to the characteristics and the design of the NILS-LSA. Participants in the NILS-LSA included randomly selected age- and sex-stratified individuals; therefore, they were equally distributed to each group by generation and sex. In contrast, most studies investigated more limited samples. The 113 healthy men in the Japanese study represented a limited sample from the same workplace and a similar younger generation. It might be that the equal proportion size of each age- and sex-stratified group in the present study led to the significant results. There is indeed a 75 % power to detect the significance for serum EPA at the highest quintile, but the DHA power is only 48 % (POWER procedure, SAS version 9.3). Most fatty acids also show low power. For fatty acids other than EPA and DHA, we consider that a failure to detect an effect was present.

The most relevant reason for the new findings in our study is that the NILS-LSA has the ability to adjust for confounding factors. Medical history including stroke and diabetes is recognised as a critical complication of depression^(^ ^38^ ^–^ ^40^ ^)^, although medical history was not adjusted for in the Japanese study mentioned above^(^ ^16^ ^)^. As many individual characteristics may be involved in the development of depressive symptoms and ageing, study results should be interpreted after adjusting for confounding factors.

There were a few differences between EPA and DHA in the present study, as the quintiles of both fatty acid concentrations showed an association with a lower OR for depressive symptoms, with significant trends. Other reports have indicated the possibility of a better effect of EPA than of DHA on depression^(^ ^41^ ^,^ ^42^ ^)^. A recent meta-analysis provided evidence that EPA may be more efficacious than DHA in treating depression^(^ ^43^ ^)^. Another meta-analysis also suggested that only regimens containing over 60 % EPA showed a highly significant effect in the treatment of depression^(^ ^42^ ^)^. DHA is also a main component of brain neurons^(^ ^44^ ^)^ and plays an important role in maintaining regular brain function^(^ ^45^ ^)^. An intervention study and a meta-analytic review indicated the possibility of a better effect of DHA than EPA on depression^(^ ^14^ ^,^ ^46^ ^)^.

This study has several limitations. First, it is not possible to prove a causal relationship between serum fatty acid concentrations and depressive symptoms because this study is a cross-sectional analysis. The association can be potentially explained by the possibility that depressive symptoms may lead to a lower intake of *n*-3 LCPUFA through decreased appetite and food consumption and a reduction in activity. Further longitudinal studies are needed to assess this relationship.

Second, we did not make a diagnosis of clinical depression in this study. Although the CES-D is a valuable assessment scale to study the relationship between depressive symptoms and several variables, it is not a clinical diagnostic tool^(^ ^20^ ^)^.

In conclusion, the present study suggests that the serum levels of EPA and DHA may be associated with depressive symptoms in Japanese with higher blood levels of *n*-3 LCPUFA. Therefore, even in countries with a higher fish intake, *n*-3 LCPUFA intake in the usual diet may lower depressive symptoms. Furthermore, the efficacy of *n*-3 LCPUFA against depressive symptoms may be present in Japanese community dwellers. Longitudinal and intervention studies are needed to elucidate any protective effects of *n*-3 LCPUFA against depression.
